# Predicting the Compressive Strength of Concrete Containing Fly Ash and Rice Husk Ash Using ANN and GEP Models

**DOI:** 10.3390/ma15217713

**Published:** 2022-11-02

**Authors:** Mohammed Najeeb Al-Hashem, Muhammad Nasir Amin, Muhammad Raheel, Kaffayatullah Khan, Hassan Ali Alkadhim, Muhammad Imran, Shahid Ullah, Mudassir Iqbal

**Affiliations:** 1 Department of Civil and Environmental Engineering, College of Engineering, King Faisal University, Al-Ahsa 31982, Saudi Arabia; 2 Department of Civil Engineering, University of Engineering and Technology, Peshawar 25120, Pakistan; 3 Department of Civil Engineering, University of Engineering and Technology, Mardan 23200, Pakistan; 4 School of Civil and Environmental Engineering (SCEE), National University of Sciences & Technology (NUST), Islamabad 44000, Pakistan

**Keywords:** compressive strength, fly ash, rice husk ash, ANN, GEP, parametric and sensitivity analyses

## Abstract

Climate change has become trending news due to its serious impacts on Earth. Initiatives are being taken to lessen the impact of climate change and mitigate it. Among the different initiatives, researchers are aiming to find suitable alternatives for cement. This study is a humble effort to effectively utilize industrial- and agricultural-waste-based pozzolanic materials in concrete to make it economical and environmentally friendly. For this purpose, a ternary blend of binders (i.e., cement, fly ash, and rice husk ash) was employed in concrete. Different variables such as the quantity of different binders, fine and coarse aggregates, water, superplasticizer, and the age of the samples were considered to study their influence on the compressive strength of the ternary blended concrete using gene expression programming (GEP) and artificial neural networking (ANN). The performance of these two models was evaluated using R^2^, RMSE, and a comparison of regression slopes. It was observed that the GEP model with 100 chromosomes, a head size of 10, and five genes resulted in an optimum GEP model, as apparent from its high R^2^ value of 0.80 and 0.70 in the TR and TS phase, respectively. However, the ANN model performed better than the GEP model, as evident from its higher R^2^ value of 0.94 and 0.88 in the TR and TS phase, respectively. Similarly, lower values of RMSE and MAE were observed for the ANN model in comparison to the GEP model. The regression slope analysis revealed that the predicted values obtained from the ANN model were in good agreement with the experimental values, as shown by its higher R^2^ value (0.89) compared with that of the GEP model (R^2^ = 0.80). Subsequently, parametric analysis of the ANN model revealed that the addition of pozzolanic materials enhanced the compressive strength of the ternary blended concrete samples. Additionally, we observed that the compressive strength of the ternary blended concrete samples increased rapidly within the first 28 days of casting.

## 1. Introduction

Different agricultural products such as wheat, sugarcane, rice, and cotton, among others, are produced in large quantities around the globe. However, the waste that is produced at the time of harvesting these crops should be studied to prevent environmental pollution. For example, a large amount of rice husk and bagasse is available after their respective utilization, and this waste must be effectively disposed of, or suitable applications for it must be found, in accordance with the principles of circular economy and sustainability. Talking of sustainability, the applications of vegetable fibers in cementitious matrix composites have also been explored by Marvila et al. [1]. The researchers observed that the plant fibers reduced the density and enhanced the water absorption capacity and tensile strength of the composites. It was also recommended by the researchers that the cellulose, lignin, and sugars present in the vegetable fibers must be removed before their application in cementitious composites. Similarly, Aquino et al. [2] used corn straw fiber in cement-lime mortars used during coating and laying blocks. It was found that corn straw fibers treated with sodium hydroxide improved the performance of specimens by demonstrating higher compressive strength and lower water absorption than the untreated fiber specimens.

Concrete is a widely used construction material with numerous benefits such as high compressive strength (CS), the ability to be cast in any desired shape, and the ready availability of its fundamental materials. The integral materials are bonded by a suitable binder, typically cement, which may or may not be convoyed with pozzolanic materials. The mechanical and durability properties of concrete depend mainly upon the gradation and physical properties of the fine and coarse aggregates used, the type of cement used, the presence of pozzolanic materials, the cement used, and the water–cement ratio [3,4,5]. The hydration of cement imparts the fusing action due to its reaction with water to form a binder gel such as calcium–silicate hydrate (C-S-H) gel and calcium–aluminate hydrate (C-A-H) gel, which are mainly used in addition to portlandite [5,6]. Different pozzolanic materials such as fly ash (FA) [7], silica fume [8], pumice (Pu) [9], blast furnace slag [10], rice husk ash (RHA) [11], and metakaolin [12] have also been used in mortar and concrete. It is important to note that pozzolans need a high-pH environment and a cation for activation to form secondary binder gels. Therefore, portlandite, produced as a result of cement hydration, is consumed by the pozzolans and results in the formation of a secondary binder gel (C-S-H, C-A-H, or C-A-S-H). The composition of secondary binder gels is highly dependent upon the chemical composition of the pozzolanic material used [13,14].

Numerous studies have shown that the partial replacement of cement with FA in the mortar and/or in the concrete improved its mechanical properties and densified its microstructure over time [15,16]. The inclusion led to a lower porosity in the samples and a higher resistance against aggressive environments. Similarly, RHA is produced by burning rice husk at around 700 °C. It is an excellent pozzolanic material and comprises a large proportion of silica, which imparts the pozzolanic property. It is important to mention here that the chemical composition and crystallinity percentage of RHA samples depends on the type of nutrients present in the soil where the crop had been sown and the burning temperature of rice husk [11,17]. Previous studies have also revealed that the partial replacement of cement with RHA improved its mechanical, durability, and microstructural properties [17]. Similarly, Pu is a natural pozzolanic material and is a type of igneous rock. It is mostly comprised of silicon dioxide in addition to other compounds such as aluminum oxide. It has been effectively used in cement mortars, improving its mechanical properties and densifying its microstructure, leading to fewer voids [18]. In addition to the use of a single pozzolanic material in mortar and concrete, the literature also reveals the influence of utilizing binary and ternary blends of different materials on different properties of concrete. For example, Tahir and Kirca [19] employed FA, SF, and blast furnace slag as ternary cementitious blends. It was observed that the ternary blend had a higher CS than the binary blend (cement and SF only). In an another attempt, Rahman et al. [20] observed that a ternary blend of MK, palm oil fuel ash, and cement improved the workability of paste, and attained high early CS with a reduced porosity in comparison with the binary blend (cement and MK only). Similarly, Anwar and Emarah [21] used a ternary blend of cement, FA, and SF to study their influence on the carbonation and ingress of chloride ions in samples. It was observed that the ternary blend improved the resistance of specimens against the ingression of these ions.

Thus, the performance of mortar and concrete is highly dependent on their constituent materials and chemical compositions. Therefore, several experimental trials must be undertaken to determine the influence of a particular constituent on the resulting properties of mortar and concrete. However, such experimental trials are arduous and time- and resource-consuming tasks [22]. Lately, artificial intelligence (AI) techniques have gained fame due to their swift learning capabilities for modeling different processes and/or phenomena, allowing models to accurately predict output(s) with the consideration of several inputs [23,24]. For instance, Baykasoglu et al. [25] made use of an artificial neural network (ANN) and gene expression programming (GEP) to forecast the CS of high-strength concrete. Topcu et al. [26] used an ANN and an adaptive neuro-fuzzy inference system (ANFIS) to forecast the CS of cement mortar containing MK. Similarly, Saridemir [27] used ANN and fuzzy logic to study the effect of MK on the CS of cement mortar. Likewise, several other models such as the radial basis function network (RBFNN), multi-layer neural networks (MLNNs) [28], decision tree models, gradient-boosting tree models [29], and extreme learning machines (ELMs) [30] have been successfully used for modeling the CS of concrete with various constituents and additives integrated. In addition to these, Nour and Mete [31] used GEP to model the ultimate strength of axially loaded, recycled-aggregate, concrete-filled steel tubular columns. It was observed that GEP successfully modelled the ultimate strength values with higher R^2^ values (0.995 and 0.996 in the training and testing phases, respectively). The researchers also provided an empirical equation to estimate the axial load capacity of tubular columns with recycled aggregate. Similarly, Gholampour et al. [32] employed GEP to predict the mechanical properties and their empirical models of concrete containing natural and recycled aggregates. A large dataset comprising 650, 421, 346, and 152 datapoints for the CS, elastic modulus, splitting–tensile strength, and flexure strength, respectively, were used to develop the model. It was observed that GEP successfully predicted the CS of concrete, as evident from the lower RMSE value of 7.8 and the coefficient of variation of 0.19.

A survey of the literature shows that different AI models have been successfully employed to model the mechanical properties of concrete containing different constituents; however, there are some problems associated with their prediction capabilities such as the production of unexpected outcomes for new datasets and the overfitting of data. Such shortcomings limit their use for forecasting in different situations. Therefore, ANN and other traditional machine learning models are considered to be black-box models [33,34]. In contrast, white-box models do not possess these limitations, and the associated information about its working and the influential variables can be extracted. One example of such models includes the gene expression programming (GEP) model, whose basic principle is based on making complex trees of chromosomes, with genes connected through linking functions; the model learns by changing their sizes and shapes [35]. Different researchers have made use of GEP models to model different properties of concrete, incorporating various materials. For instance, a study led by Iqbal et al. [36] used GEP to model the mechanical properties of green concrete with waste foundry sand integrated. GEP has also been successfully used to model the resilient modulus of stabilized soils [37]. The use of GEP algorithms has enabled researchers to accurately predict the output (R^2^ > 0.85), and at the same time, provide an empirical equation for the output in terms of the input variables.

Considering the ability of different AI models to predict the properties of concrete, this study aimed to perform a comparative analysis of the ANN and GEP models to predict the compressive strength of ternary blended concrete to avoid laborious and time- and resource-consuming experimentation. The ANN and GEP models were utilized to model the compressive strength of ternary blended concrete using different input variables such as the amounts of cement, fine aggregate, coarse aggregate, water, superplasticizer, fly ash, and rice husk ash, and the age of the sample. In addition to the performance comparison, we took advantage of the white-box nature of the GEP model to derive an empirical equation for the compressive strength of ternary blended concrete in terms of the above-mentioned inputs. Finally, parametric analysis was also conducted to study and understand the influence of the different input parameters on the compressive strength of ternary blended concrete. These analyses will be a significant contribution to the field of civil engineering materials as it employs both black-box and white-box AI models to predict the compressive strength of concrete by incorporating two different pozzolanic materials and comparing their performance.

## 2. Methodology

### 2.1. Database Description and Statistics

**Figure 1:**
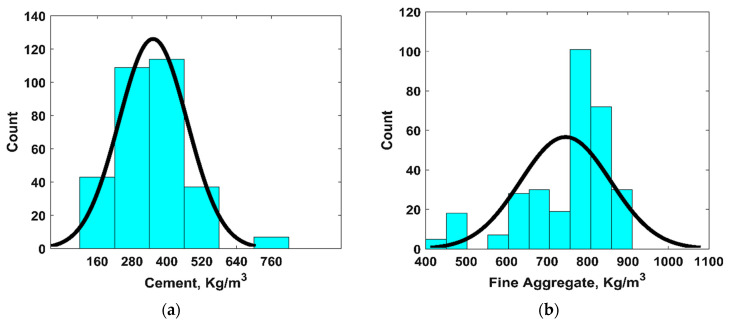

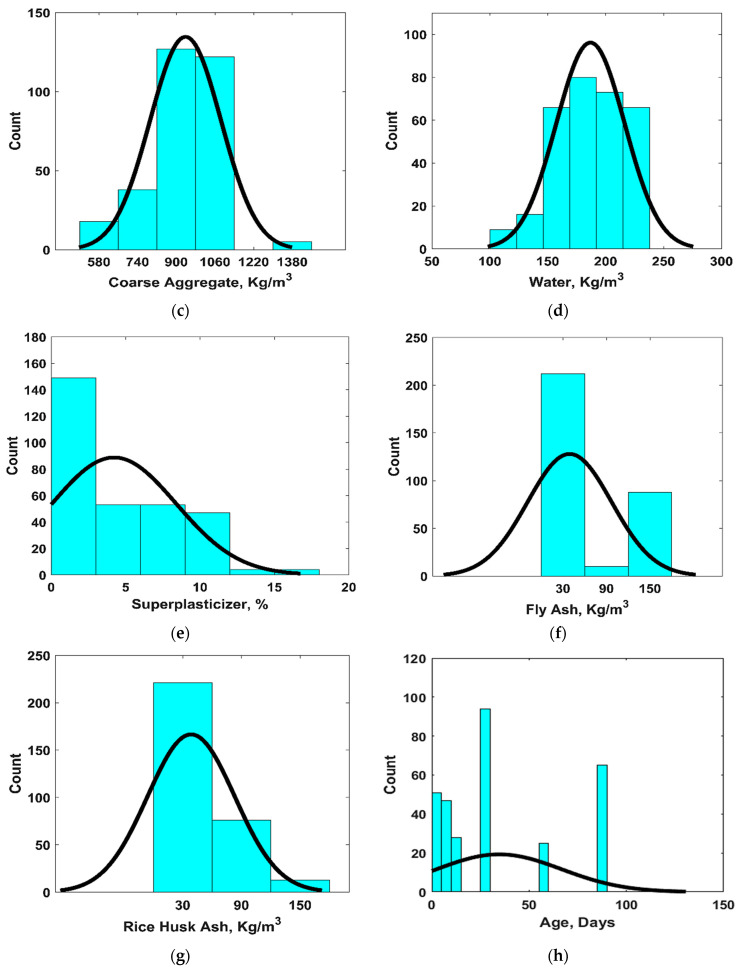
Frequency histograms of input variables: (**a**) cement; (**b**) fine aggregate; (**c**) coarse aggregate; (**d**) water; (**e**) superplasticizer; (**f**) fly ash; (**g**) rice husk ash; (**h**) age.

In order to model the CS of ternary blended concrete using GEP, a database comprising the input and output parameters is required. For this purpose, one could use one’s own experimental database or an aggregation of the results from past research articles, published in well-reputed journals. The latter approach was adopted for this study and, upon a survey of the literature, it was revealed that none of the articles had the correct ternary blend (cement, RHA, and fly ash); therefore, the articles with a binary blend (cement and one pozzolan) and a ternary blend (cement and two pozzolans) were explored, comprising one or two of the above-mentioned materials. As a result of this effort, a database was prepared from the studies conducted by Iftikhar et al. [38], Ozcan and Emin [18], and Saridemir [39]. Our database was comprised of 310 data points with nine input parameters, viz. the amount of cement, the fine aggregate (F.ag.), the coarse aggregate (C.ag.), the water, the superplasticizer (SP), the FA, the RHA, the age of the sample, and one output (i.e., CS). The AI models, which will be trained and validated using the above-mentioned input parameters, will be able to predict the CS of ternary blended concrete. However, it is pertinent to mention here that the values of the input parameters must be within the range at which the AI models have been trained and validated. For this purpose, the descriptive details of the dataset are presented in Table 1 including the minimum and maximum values of the input parameters. Similarly, Figure 1 shows the frequency histograms of the input parameters. It can be inferred from Figure 1 and the kurtosis values in Table 1 that the distribution of several input parameters such as the amount of cement, F.ag., C.ag., and RHA has a sharp peak. Similarly, the negative values of skewness in Table 1 also confirms the presence of a fatter tail in the distribution curves of F.ag., C.ag., and water on the left side of Figure 1. Similarly, Table 2 and Table 3 present the physical properties and chemical composition of ordinary Portland cement, RHA, and FA.

### 2.2. Tuning of Hyperparameters during GEP Modeling

**Figure 2:**
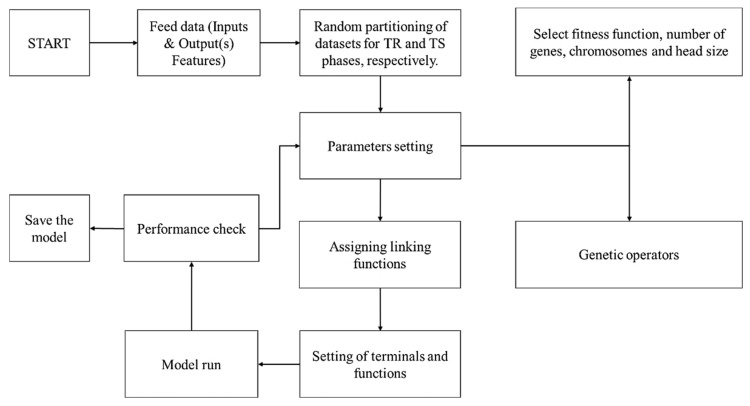
Flowchart of GEP modeling.

GeneXprotools was employed for the successful development and training of the GEP models. The process involved retrieving the data into its interface and dividing the attributes into input and output variables. Similarly, the dataset was also divided into two subsets, namely the training dataset and the testing dataset. The dataset was split into the aforementioned subsets in a 70/30 percentage. As a result, 70% of the dataset (217 data points) was used for the training (TR) phase and the remaining 93 data points were used for the testing (TS) phase. The next step involved setting the parameters of the GEP model. For this reason, the number of chromosomes (N_c_) varied from 30 to 200, while the head size (H_s_) varied from 8 to 12. Similarly, the number of genes (N_g_) also plays a vital role in improving the performance of the GEP model. For this purpose, three distinct numbers of genes (i.e., 3, 4, and 5) were used to evaluate their effect on the performance of the models. The addition function was used as a linking function among genes. This was selected after a rigorous exercise, which involved exploring numerous linking functions (+, −, ×, /) among the genes. The root mean square error (RMSE) was used as the cost function. The flowchart of GEP modeling is shown in Figure 2, while Table 4 provides the values of the hyperparameters for the best-performing GEP model.

Usually, a trial-and-error-based approach is adopted for setting the parameters of GEP models. Previously, researchers used to program GEP algorithms to randomly select the datapoints for the TR and TS datasets. As a result of this practice, the developed models used to overfit the data during the TR process, with a subsequent improvement in their performance in the TS phase; however, this practice led to a reduction in the performance of the validation data. This problem was resolved by the approach followed by Gandomi and Roke [35], who selected a model with a minimum objective function (OF) [36]. The values of the OF ranged between 0 and the maximum value, and models with an OF value approaching zero were considered to be better models. Similarly, different performance indices such as the coefficient of determination (R^2^), the RMSE, and the mean absolute error (MAE) were employed for assessing the performance of the proposed models. The ideal values of these statistical indices are presented in Table 5.

In order to determine the best-performing model, the hyperparameters of the GEP model were tuned a number of times (trial-and-error-based approach). As a result, a number of models were developed with different numbers of chromosomes, different numbers of genes, and different head sizes. At first, the N_c_ values were changed from 30 to 200, while keeping the H_s_ (i.e., 8) and the N_g_ (i.e., 3) values constant. An optimal model performance was obtained when N_c_ = 100. Similarly, the H_s_ value was changed from 8 to 12, keeping the other two variables constant, in order to obtain an optimal model performance for a particular value of H_s_. A similar procedure was followed to determine the optimal number of N_g_. As a result of the hyperparameter tuning process, the N_c_, H_s_, and N_g_ values for an optimally performing GEP model were determined to be 100, 10, and 5, respectively. Table 4 presents the details of the performance of the optimum model, obtained as a result of this tuning process.

### 2.3. Artificial Neural Network Modeling

An ANN is a soft-computing algorithm that simulates the biological neural networks of learning algorithms. Its architecture comprises an input layer, a hidden layer, and an output layer. The input layer is comprised of non-computational neurons, which are equal to the number of input variables, while the output layer has computational neurons equaling the number of target variables. The number of neurons in the hidden layer depends on the trial, which yields the best performance. The neurons in the input layer receive information from outside source(s) in terms of inputs, whereas the computational neurons conduct linear and nonlinear operations on the input data. Every neuron combines the weighted values from the related input neuron obtained in the linear phase. Additionally, an activation function is added before the outcome can be transmitted as an output. Similarly, the neurons of the hidden layers are linked together by nodes with different weights. These are used to connect the non-computing and computing neurons. The number of neurons in the input layers depends upon the number of influential features, while the number of neurons in the output layer is equal to the number of output variable(s). The Levenberg–Marquardt back-propagation algorithm was used to train the models. The number of neurons in the hidden layers was changed from 8 to 12 and the optimum performance was obtained with 10 neurons [43,44,45,46].

## 3. Results and Discussion

### 3.1. Performance of the Models

The performances of the GEP and ANN models were assessed using different approaches such as statistical evaluation, slope of regression line, and the predicted–experimental (P/E) ratio.

#### 3.1.1. Statistical Evaluation

As discussed previously, different statistical indices were employed to judge the performance of both of the models. The reason for adopting other indices such as RMSE and MAE was that a higher value of R^2^ indicates a good agreement between the predicted and actual values. However, determining the performance of a model based on “R^2^” alone is not sufficient, and other statistics must also be considered. In this regard, this study calculated the values of RMSE and MAE to evaluate the performances of the models, in addition to the R^2^ values, as shown in Table 4. The same indices were employed to rank the performances of these models, as shown in Table 6. It is evident from Table 6 that the ANN model performed better, as shown by their higher R^2^ (0.94 and 0.88) and lower RMSE (6.23 and 8.27) and MAE (4.01 and 6.07) in the TR and TS phases, respectively. The performance indices also reveal that the performance of the ANN model was slightly reduced in the TS phase.

#### 3.1.2. Regression Slopes Analysis

The analysis of regression slopes is a useful technique for evaluating the performance of a model against the experimental/actual values. The theoretical/experimental/actual values are plotted on the *x*-axis, while the predicted values are plotted on the *y*-axis. The slope of the line between the actual and forecasted values is noted. This assessment method was employed here and the regression slope lines were plotted for the best-performing GEP and ANN models. It is important to reiterate that an ideal line, with a slope equal to one, will make an angle of 45° with the *x*-axis. The performance of the model will be excellent (i.e., the predicted values will be closer to the actual values), if the plotted points lie close to the standard line. A regression line whose slope value is closer to 1 and with correlation values ≥0.8 will have minimal values for the error indices [47,48].

**Figure 3:**
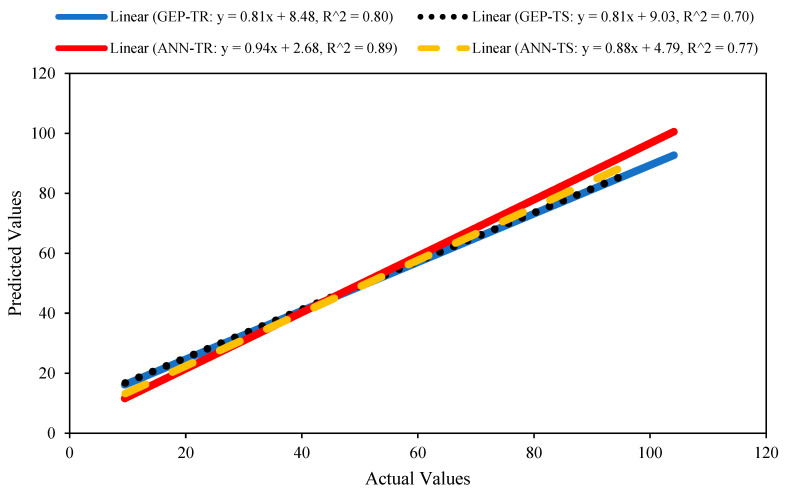
Comparison of the regression slopes for the best-performing GEP and ANN models.

Figure 3 shows the comparison of regression slopes for the best-performing GEP and ANN models for the TR and TS phases, respectively. It is evident from Figure 3 that the value of R^2^ was higher for the TR dataset (i.e., 0.80 and 0.89 for the GEP and ANN models, respectively), while higher slope values were observed for the ANN model (0.94 and 0.88 during the TR and TS phases, respectively) than for the GEP model. Similarly, Figure 3 demonstrates that the value of the slope for the ANN model was reduced in the TS phase (m = 0.88) from m = 0.94 in the TR phase.

#### 3.1.3. Model of Predicted–Experimental Ratio

**Figure 4:**
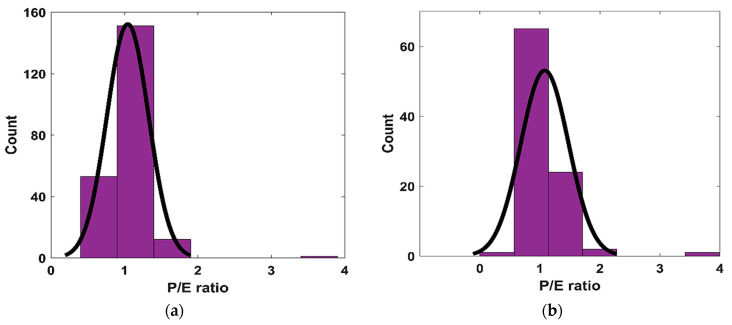

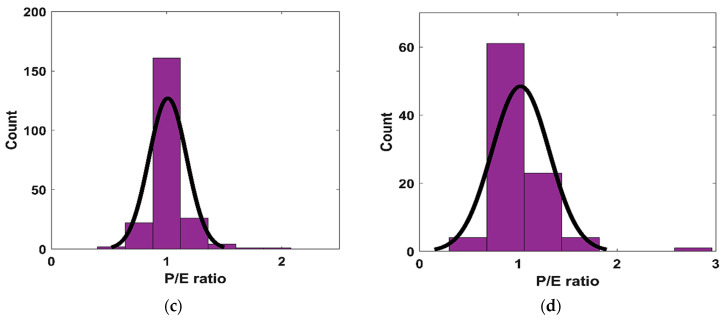
The P/E ratio distribution: (**a**) GEP-TR phase, (**b**) GEP-TS phase, (**c**) ANN-TR phase, and (**d**) ANN-TS phase.

The performances of the different models, produced through different trials, were investigated using the P/E ratio. Figure 4 depicts the P/E ratios for the best-performing GEP and ANN models for both the TR and TS phases. Figure 4a,b shows that a higher number of counts could be observed for the P/E ratio around 1 for the GEP model during the TR and TS phases. However, there were a few counts of the P/E ratio exceeding 3 for the GEP model during both phases. Similarly, the ANN model performed comparatively better, and a larger count for the P/E ratio values equaling one could be observed during both phases; however, some higher values (i.e., P/E ratio >2) could also be observed for the ANN model during the TS phase, as evident from Figure 4d. This is another visual justification for the ANN model performing better than the GEP model in accurately predicting the CS values of ternary blended concrete; the values derived by the ANN model were in good agreement with the experimental values.

Table 7 presents the comparison of the currently proposed models for predicting the CS of ternary blended concrete with previous studies. It is important to note here that the CS of binary blended concrete has successfully been modeled using different AI models in the past (Table 7). For example, Song et al. [49] modeled the CS of binary blended concrete (cement and FA) using GEP and ANN. He observed that the GEP model was more robust than the ANN model by securing a higher R^2^ of 0.86. The current study modeled the CS of ternary blended concrete satisfactorily while considering two different pozzolanic materials (i.e., RHA and FA).

Considering the excellent performance of the proposed AI models (especially ANN), as demonstrated in Section 3.1.1, Section 3.1.2 and Section 3.1.3 of this manuscript, it is without any doubt that the proposed models can predict the CS of ternary blended concrete accurately. The statistical analysis in the preceding sections revealed that the ANN model performed better (higher R^2^ and lower RMSE and MAE values), however, due to its black-box nature, no empirical equation could be derived for the CS of the ternary blended concrete. In contrast, the performance of the GEP model was slightly poorer than the ANN model, however, it could provide an empirical equation for future use. The empirical equation as discussed in Section 3.2 is capable of calculating the CS of ternary blended concrete as a function of the considered input parameters. However, it is important to recall here that these AI models can work efficiently and accurately predict the CS of ternary blended concrete only if the values of the input parameters lie within the range (i.e., between the minimum and maximum values of input parameters, as illustrated in Table 1). Similarly, the models will not work accurately if the mix design of ternary blended concrete contains other admixtures and/or additives.

### 3.2. GEP Formulations

**Figure 5:**
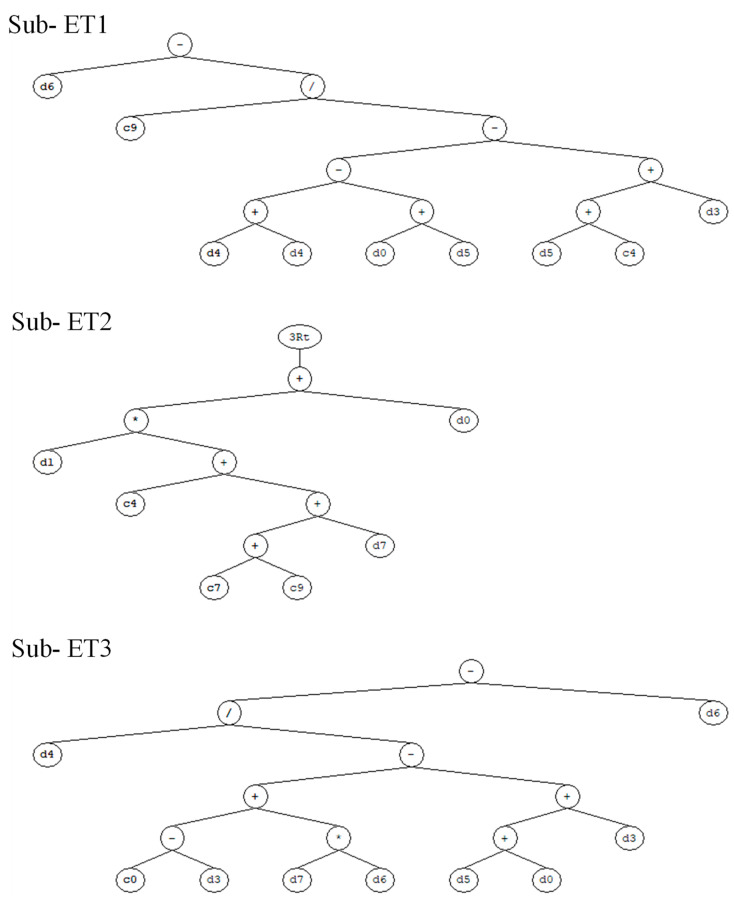

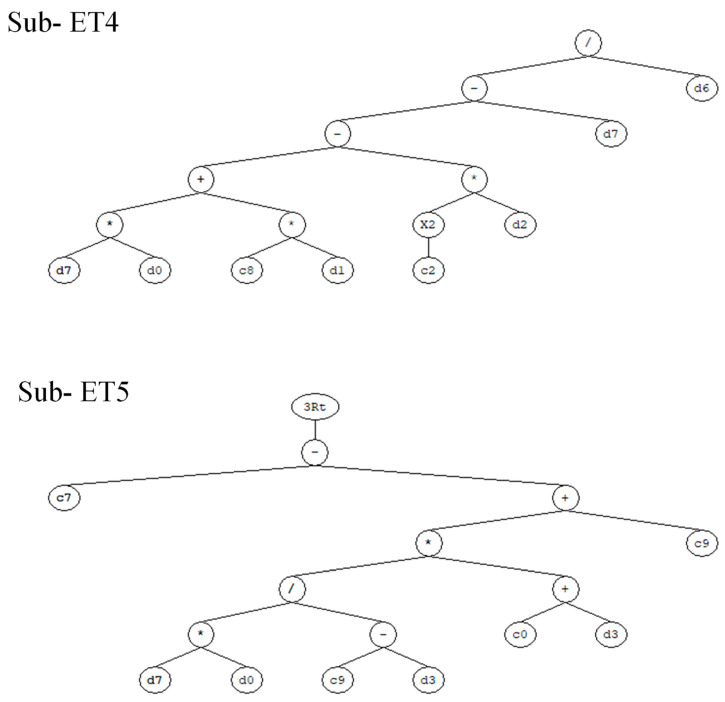
Expression tree obtained from the optimized GEP model.

GEP, being a white-box model, enables researchers to derive empirical equations for the intended output in terms of the influential input variables. This can be achieved by utilizing the expression tree and the GEP model of the best-performing model. Considering this advantage, the expression tree (Figure 5) and the GEP model of the optimally performing GEP model were utilized in order to obtain an empirical equation for the CS of ternary blended concrete. This equation can be further used for the sensitivity and parametric analysis of input variables. Equation (1) presents the expression for computing the CS of ternary blended concrete in terms of the input variables, viz., the amount of cement, the fine aggregate, the coarse aggregate, the water, the superplasticizer, the fly ash, the rice husk ash, and the age of the sample.

(1) CS=A+B+C+D+E

where

(2) $$A=Water-\frac{11.67}{2\times F.ag.-SP-2\times C.ag.-0.24+RHA}$$

(3) $$B={\left(\left(Cement\times \left(-1.02+Age\right)\right)+SP\right)}^{\frac{1}{3}}$$

(4) $$C=\frac{F.ag.}{\left(-8.37-RHA\right)+\left(Age\times Water\right)-\left(C.ag.+SP+d4\right)}-Water$$

(5) $$D=\frac{\left(\left(Age\times SP\right)+\left(12.37\times Cement\right)-\left(42.51\times FA\right)\right)-Age}{Water}$$

(6) $$E={\left(1.59-\left(\frac{Age\times SP}{9.49-RHA}\right)\times \left(-8.39+RHA\right)+9.49\right)}^{\frac{1}{3}}$$

It is important to mention here that, while using the above equation for computing the CS of ternary blended concrete, the amount of cement, F.ag., C.ag., and water must be expressed in Kg/m^3^; superplasticizer and fly ash and rice husk ash must be expressed by the percentage of binder used (i.e., cement). Similarly, age must be expressed in days.

### 3.3. Parametric Analysis

Parametric analysis, also called monotonicity analysis, is normally carried out to authenticate the reliability of AI models. For this purpose, its performance is evaluated based on the simulated datasets. Similarly, sensitivity analysis can also be performed for such purposes, as it illustrates the response of the prediction model to the variation in the input parameters [37,51]. Therefore, a parametric analysis was conducted in this study to assess the influence of each input feature on the CS of the ternary blended concrete specimens.

**Figure 6:**
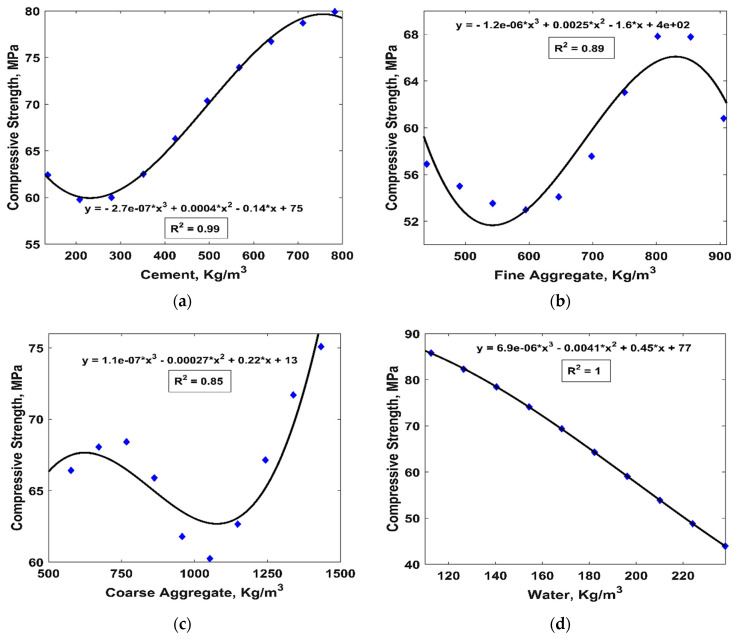

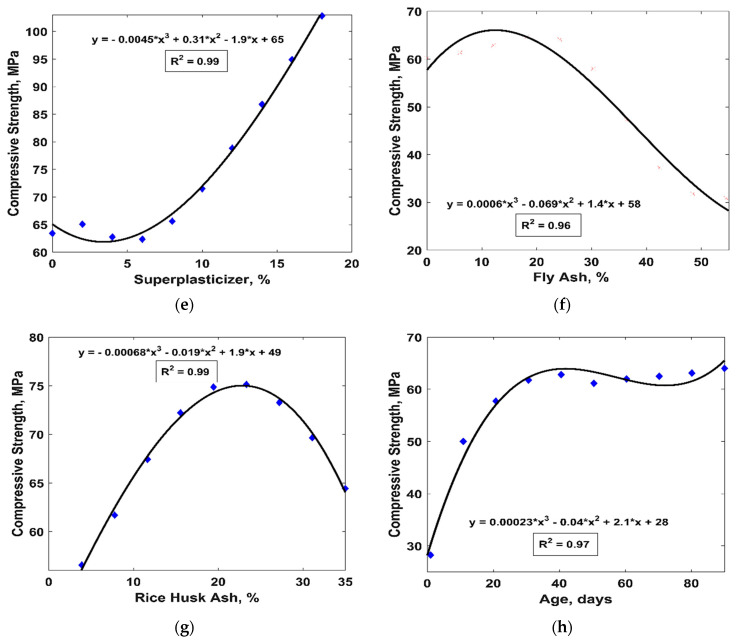
The parametric analysis of the input variables: (**a**) cement; (**b**) fine aggregate; (**c**) coarse aggregate; (**d**) water; (**e**) superplasticizer; (**f**) fly ash; (**g**) rice husk ash; (**h**) age.

Parametric analysis of all of the input variables (cement, F.ag., C.ag., water, SP, FA, RHA, and age) was carried out using the ANN model (the ANN model was chosen due to its superior performance in comparison with the GEP model) in order to assess their influence on the resulting CS of the concrete specimens. Table 8 displays the possible combinations of the different input parameters adopted for the parametric analysis. The analysis was executed by changing one input parameter from its minimum value to its maximum value while maintaining the rest of the input variables at their average values. For example, the CS values of the concrete specimens were evaluated by varying the amount of cement from its minimum value to its maximum value while maintaining the rest of the input variables at their mean values. This practice was performed for all of the input variables. Figure 6 illustrates the variation in the CS values in response to the changes in each input variable, as per the above-described procedure. It is clear from Figure 6a–c,e,h that the CS improved with the amount of cement, fine aggregate, coarse aggregate, superplasticizer, and the age of the sample. Similarly, Figure 6f,g shows that the CS of the ternary blended concrete samples improved with the addition of fly ash and rice husk; however, their addition in higher replacement amounts led to a decrease in CS. These findings are in accordance with previous studies because both the cement and the pozzolanic materials contribute toward primary and secondary binder gel (C-S-H, C-A-H and C-A-S-H) formation, and because pozzolanic materials need time, a sufficient amount of portlandite, and a high pH value for their activation; these factors subsequently increase their contribution to strength [52,53]. Similarly, the application of superplasticizer improves the workability of the mix, thereby allowing for better compaction of the sample with reduced air voids and higher density. Furthermore, Figure 6d demonstrates that the CS decreased with the amount of water. This is supported by the fact that only a reasonable amount of water is needed for cement hydration and subsequent pozzolanic reaction [13]. Similarly, Figure 6h shows that the rate of increase in CS was higher during the initial 28 days and was subsequently decreased with a further increase in age (beyond 28 days of age).

## 4. Conclusions

This study was performed with the aim of effectively utilizing fly ash, an industrial waste, and rice husk ash, an agricultural waste, in concrete to understand their influence on the compressive strength of concrete using gene expression programming and artificial neural networks. Different variables such as the quantity of cement, fly ash, rice husk ash, fine and coarse aggregates, water, and superplasticizer and the age of the samples were considered to determine their influence on the compressive strength of the ternary blended concrete samples. The main conclusions drawn from this study are as follows:

1. The GEP model with the optimal performance was obtained with 100 chromosomes, a head size of 10, and five genes. This model showed high R^2^ values of 0.80 and 0.70, low RMSE values of 8.52 and 9.30, and low MAE values of 6.31 and 7.38 in the TR and TS phases, respectively.
2. The regression slopes analysis revealed that the predicted values produced by the ANN model were in good agreement with the experimental values, as evidenced by its higher R^2^ values (0.89 and 0.77 in the TR and TS phases, respectively). Similarly, the P/E ratio analysis revealed that the ANN model performed better than the GEP model, with a larger frequency observed for the P/E ratio equaling one during both the TR and TS phases; however, during the TS phase, a small number of counts were observed for the P/E ratio >2.
3. Similarly, a parametric analysis of the best-performing model (the ANN model) showed that the compressive strength of the ternary blended concrete samples improved with the amount of pozzolanic materials and superplasticizer added and the age; therefore, the experimental observations were confirmed. It was also verified that the amount of water plays a prominent role in controlling the compressive strength of concrete; attention must be paid to decisions surrounding the amount of water and the workability requirements in order to achieve the desired strength of a given sample. Finally, it was observed that the compressive strength of the ternary Yes blended concrete samples enhanced rapidly within the first 28 days of casting; after this period, the strength gain rate reduced slightly due to pozzolanic reactions.

## 5. Future Work

It is recommended that the proposed AI models trained on previous research studies are also validated by conducting new experimental trials incorporating these waste materials and their prediction performance can be judged. Similarly, the influence of these waste materials on other mechanical properties such as the split-tensile strength and modulus of elasticity of concrete can also be modeled using these AI techniques.

## Figures and Tables

**Table 1:** Descriptive statistics of the input variables.

Descriptive Statistics	Cement (Kg/m^3^)	F.ag. (Kg/m^3^)	C.ag. (Kg/m^3^)	Water (Kg/m^3^)	SP, %	FA, % (Kg/m^3^)	RHA, % (Kg/m^3^)	Age, Days	CS, MPa
Mean	352.40	745.73	938.38	186.90	4.22	10.95 (39.16)	0.92 (38.60)	34.56	43.29
Standard Error	6.68	6.32	8.35	1.68	0.24	0.94 (3.29)	0.23 (2.53)	1.82	1.04
Standard Deviation	117.70	111.30	146.90	29.58	4.17	16.49 (57.97)	4.08 (44.57)	32	18.32
Sample Variance	13,849	12,401	21,603	874	17.42	272.10 (3361)	16.71 (1986)	1023	335
Kurtosis	2.33	0.92	1.58	−0.36	−0.52	−0.90 (−1.27)	21.32 (0.09)	−0.80	0.42
Skewness	1.01	−1.22	−0.32	−0.20	0.68	0.94 (0.80)	4.66 (0.93)	0.82	0.67
Minimum	136.10	439.60	576.90	112.50	0	0	0	1	9.49
Maximum	783	905.40	1433.50	238	18	54.50 (168.3)	25 (171)	90	104.10

**Table 2:** Physical properties of cement, rice husk ash, and fly ash.

Material	Specific Gravity	Blaine’s Fineness (m^2^/g)
Cement	3.15 [17]	0.36 [17]
Rice Husk Ash	2.83 [17]	0.62 [40]
Fly Ash	2.22 [41]	>0.38 [42]

**Table 3:** Chemical composition of cement, rice husk ash, and fly ash.

Ordinary Portland Cement [13]		Rice Husk Ash [17]		Fly Ash [13]	
Compound	Percentage	Compound	Percentage	Compound	Percentage
SiO_2_	21.00%	SiO_2_	96.11	SiO_2_	40.00%
Al_2_O_3_	6.00%	Al_2_O_3_	-	Al_2_O_3_	26.32%
Fe_2_O_3_	2.58%	Fe_2_O_3_	0.39	Fe_2_O_3_	15.16%
CaO	60.02%	CaO	1.03	CaO	7.83%
SO_3_	9.30%	SO_3_	0.21	SO_3_	6.14%
TiO_2_	0.29%	TiO_2_	0.03	TiO_2_	2.55%
K_2_O	0.81%	-	1.16	K_2_O	1.41%

**Table 4:** Performance of the optimal GEP and ANN models.

Model	No. of Variables	No. of Chromosomes	Head Size	No. of Genes	TR Phase			TS Phase		
					R^2^	RMSE	MAE	R^2^	RMSE	MAE
GEP	8	100	10	5	0.80	8.52	6.31	0.70	9.30	7.38
ANN	-	-	-	-	0.94	6.23	4.01	0.88	8.27	6.07

**Table 5:** Ideal values of the performance indices.

Index	Range/Ideal Value
R^2^	(0–1)/1
RMSE	(0–∞)/0
MAE	(0–∞)/0

**Table 6:** Ranking of models based on R^2^ and RMSE.

Statistic	R^2^		RMSE		MAE	
Rank	1st	2nd	1st	2nd	1st	2nd
TR and TS Phase	ANN	GEP	ANN	GEP	ANN	GEP

**Table 7:** A comparison of the proposed model performance with previous studies.

Research Study	Binder(s)	AI Model	R^2^
Iftikhar et al. [38]	Cement and RHA	GEP and Random Forest Regression (RF)	GEP = 0.96 and RF = 0.91
Song et al. [49]	Cement and FA	GEP and ANN	GEP = 0.86 and ANN = 0.81
Salami et al. [50]	Cement, FA, and furnace slag	Least square support vector machine (LSSVM), GEP, ANN and RF	LSSVM = 0.95, GEP = 0.89, ANN = 0.91, and RF = 0.86
This study	Cement, RHA, and FA	GEP and ANN	GEP = 0.70 and ANN = 0.77

**Table 8:** Dataset used for the parametric analysis.

Input Variables		Constant Input Parameters	No. of Data Points
Parameter	Range		
Cement	136.1–783	F.ag. = 745.74, C.ag. = 938.37, Water = 186.88, SP = 4.22, FA = 39.16, RHA = 0.92, Age = 34.56	10
F.ag.	439.59–905.4	Cement = 352.44, C.ag. = 938.37, Water = 186.88, SP = 4.22, FA = 39.16, RHA = 0.92, Age = 34.56	
C.ag.	576.88–1433.50	Cement = 352.44, F.ag. = 745.74, Water = 186.88, SP = 4.22, FA = 39.16, RHA = 0.92, Age = 34.56	
Water	112.5–238	Cement = 352.44, F.ag. = 745.74, C.ag. = 938.37, SP = 4.22, FA = 39.16, RHA = 0.92, Age = 34.56	
SP	0–18	Cement = 352.44, F.ag. = 745.74, C.ag. = 938.37, Water = 186.88, FA = 39.16, RHA = 0.92, Age = 34.56	
FA	0–168.3	Cement = 352.44, F.ag. = 745.74, C.ag. = 938.37, Water = 186.88, SP = 4.22, RHA = 0.92, Age = 34.56	
RHA	0–25	Cement = 352.44, F.ag. = 745.74, C.ag. = 938.37, Water = 186.88, SP = 4.22, FA = 39.16, Age = 34.56	
Age	1–90	Cement = 352.44, F.ag. = 745.74, C.ag. = 938.37, Water = 186.88, SP = 4.22, FA = 39.16, RHA = 0.92	

## Data Availability

The data used in this research have been properly cited and reported in the main text.
